# A computational model of epidemic process with three variants on a synthesized human interaction network

**DOI:** 10.1038/s41598-024-58162-z

**Published:** 2024-03-29

**Authors:** Seprianus, Nuning Nuraini, Suhadi Wido Saputro

**Affiliations:** Department of Mathematics, Institut Teknologi Bandung, Bandung, Indonesia

**Keywords:** Virus variants, Human interaction network, Microscopic epidemic, Mathematics and computing, Epidemiology

## Abstract

Virus mutations give rise to new variants that cause multiple waves of pandemics and escalate the infected number of individuals. In this paper, we develop both a simple random network that we define as a synthesized human interaction network and an epidemiological model based on the microscopic process of disease spreading to describe the epidemic process with three variants in a population with some features of social structure. The features of social structure we take into account in the model are the average number of degrees and the frequency of contacts. This paper shows many computational results from several scenarios both in varying network structures and epidemiological parameters that cannot be obtained numerically by using the compartmental model.

## Introduction

All viruses change over time through mutations and give rise to new variants. Although most of them have little biological significance, a small number of them, called ’variants of concern’, appear to cause multiple waves of pandemic and escalate the infected number of individuals^1,2^. New variants not only result in increased transmissibility, morbidity, and mortality but also cause reinfection in previously infected and recovered individuals, and ineffectiveness of vaccines^3^. For example, SARS-CoV-2 the virus causing COVID-19 emerged originally in Wuhan, China, in December 2019. Several novel variants of SARS-CoV-2 emerged and took over the world since then. The alpha variant was initially detected in the UK in September 2020 and is more transmissible than the original strain. The beta variant was detected in South Africa in November 2020 and is more transmissible than previous strains. It can reinfect those infected with the original strain. The gamma variant was detected in November 2020 in Brazil. It can also reinfect and be more infectious. Delta variant was initially detected in India in October 2020. It is more transmissible, infectious, and lethal^4,5^. Omicron variants were first detected in November 2021 in Africa and since December 2021 confirmed cases have been reported from many countries in Europe^6^. To date, caused by COVID-19, more than 590 million confirmed cases and at least 6.5 million deaths have been reported worldwide^7^. Although the virus is less fatal than it was in 2020, it is likely to continue to undergo mutation and produce future variants. It forces all countries in the world to enter the endemic stage of COVID-19^8^.

Virus mutation is natural to occur. Once a virus invades a host (human or animal), it replicates itself to produce more viruses. In this process, some errors occur. We call the error a mutation and new variants are expected to rise^9^. As a consequence, we can not stop the virus from mutating as long as the virus infects^10,11^. Unfortunately, in this current era, globalization has made the world interconnected. It increases the amount, frequency, and speed of population mobility and in turn, causes the range of disease spread to become borderless. Every person in any country becomes vulnerable when a pandemic attacks a certain region. As a result, infectious diseases caused by mutating viruses spread over the world at an unprecedented frequency and speed^12^.

Nowadays, although vaccination is the most effective way to control the epidemic spreading^13^, no vaccine is $100\%$ effective in the prevention of the disease spread^3^. Moreover, when the vaccination rate is low both in production and administration, this provides enough time for the virus to mutate and become vaccine-resistant^14^. As a consequence, the emergence of new variants can not be prevented^15^. The low rate of vaccination is caused by many factors such as uneven distribution of vaccination, and a lack of consideration of vaccination scenarios^16^.

Most epidemiological models use a compartmental model to describe the epidemic process where each individual is supposed to be one of the compartments such as susceptible (S), infected (I), or recovered (R), and commonly follow a general susceptible-infected-recovered (SIR) model^17^. However, most of these models assume that the population is “fully mixed’ or “mass-action approximation”, meaning that every individual has an equal chance, per unit of time, of coming into contact with every other member of the population they belong^18,19^. As a result, these models are not a realistic representation of the disease spread in a human population. Since in practice, each individual has a finite set of connections and frequency of contacts that the infection can pass through. It means that an infected individual does not have an equal probability of infecting a small number of others in the population. Therefore, a network will be used to capture the human interactions and how the disease spreads at the individual level or microscopic scale.

Networks and the epidemiology of directly transmitted infectious diseases are fundamentally linked^20^. As we said above each individual in a human population has a finite set of connections and the intensity of interactions, meaning there are certain connections among individuals in the population that can be a structure of human interaction. The structure underlying human interaction can be captured by a network. In a network, all individuals in the population can be represented by a set of nodes or vertices, and connections among them can be represented by edges. The number of edges incident to a node is called the degree of a node and the weight of an edge can represent the intensity of interaction between two nodes.

The epidemic model with two variants using the compartmental model has been studied analytically^2^. It shows that the cumulative number of infected individuals of two variants can be well approximated by independent logistic functions. It also shows that the model can validate empirically the multiple waves of the COVID-19 pandemic in some countries. The study of two variants of the epidemic by performing a numerical simulation of the microscopic model on the networks has been done^21^. In the study, a new variant is added in the middle of a simulation. As a result, the infectivity of variants determines the epidemic size. When a highly infectious variant is added, the variant spreads quickly. The network structure also stimulates the rapid increase of infection.

Human interactions in a population can be represented as a contact network where each individual is regarded as a node and interaction between two individuals can be considered as an edge. The intensity of interaction between two nodes can be regarded as a weight given to the edge. In this study, the weight we give to each edge in the network is a natural number that represents how many times two connected individuals interact in proximity of each other in one day. This is what we call contact which is defined as a two-way conversation between two individuals in a certain range of distance such that possible for an infection to transmit^22^.

In this paper, we first generate a synthesized human interaction network with a given average degree and random frequency of contacts. Then, we develop a mathematical model that can capture the microscopic process of disease spreading in the network. At the initial time, we choose randomly $N_{0}$ individuals to get infected by Variant 1. Variant 2 will emerge from infections of Variant 1. It is described by randomly moving an infected individual due to Variant 1 to the infected individual due to Variant 2 with a certain probability. Likewise, Variant 3 will emerge from the infection of Variant 2, and Variant 2 mutates to Variant 3 with similar procedures.

With the varying average degrees and a certain range of contact frequency, we will show that the epidemic size is not only determined by varying the epidemiological parameters but also by a complex combination with the network structures. By varying the network structure, we can accommodate the mitigation strategies to suppress the spread of the disease when no vaccine and medicine are administered.

However, this study has some limitations. The model we will develop relies heavily on computation and strongly depends on the real data of social contacts. The simulation we have performed is limited to 10, 000 individuals and in turn, the network we generate with this number of individuals does not represent the real social network in a big population. In addition, the real data on social contact is not easy to collect and has also not been provided yet. Therefore, we limit our model applications to smaller-scale environments such as campuses, schools, work environments, and public places as theoretical modeling and simulations. We also agree for ethical reasons that the process of the spread of infectious disease can never be experimentally studied in our society^23^. This paper is just proposed to provide decision-makers with the predictive power of the epidemiological models with more than one variant in a smaller population from theoretic simulations.

## Results

In this section, we will show some simulation results obtained from the model we developed in the “Method” Section. We perform each simulation with 100 samples for $t_{f}=400$ days. We set the total number of nodes $N=10,000$ and the initial condition $N_{0} =5$. By varying the average degree, the range of contact frequency, and some epidemiological parameters, we can obtain the various scenarios. We can also obtain abundant results by only varying the epidemiological parameters when the network structure is fixed. However, in this simulation, we want to show how the network structure greatly affects the dynamics of disease spread in the network with fewer variations in the value of epidemiological parameters. We want to show that mitigation strategies like containment measures can be considered as variations of the average degree and contact frequency. To simplify our choice in simulations, we also set the average duration of the infectious period $\tau _{v}=15$ days for each variant. Here, we chose only four types of scenarios based on our assumption when some containment measures are implemented in the population during the pandemic. The four types of scenarios are the following:

1. Epidemic model with three variants on the synthesized human interaction network without any containment measure. We assume that ${\bar{k}}=7$ and $\omega =5$.
2. Epidemic model with three variants on the synthesized human interaction network with self-quarantine but no physical distancing. We assume that ${\bar{k}}=3$ and $\omega =5$.
3. Epidemic model with three variants on the synthesized human interaction network with no self-quarantine but with physical distancing. We assume that ${\bar{k}}=7$ and $\omega =1$.
4. Epidemic model with three variants on the synthesized human interaction network with self-quarantine and physical distancing. We assume that ${\bar{k}}=3$ and $\omega =1$.

### Scenario 1

**Figure 1:**
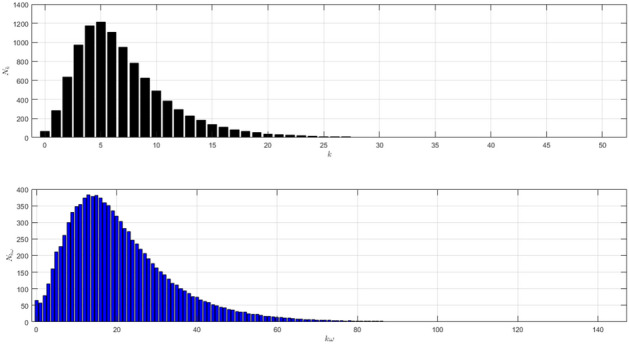
The network structure of Scenario 1. The black histogram shows the degree distribution of the network with ${\bar{k}}=7$ and $\omega =5$. It shows that the network has a lot of hubs and fewer isolated nodes. The blue histogram shows the contact distribution. More than half of the population have a contact frequency of $20-90$.

The network structure of Scenario 1 with ${\bar{k}}=7$ and $\omega =5$ is shown in Fig. 1. The black histogram shows the degree distribution of the network. The blue one shows the contact distribution of the network. We can refer to the nodes with high degrees as hubs^24^. We will call the node a hub if its degree $k\ge 14$. The role of hubs in the epidemic process has been studied in^25^.

**Figure 2:**
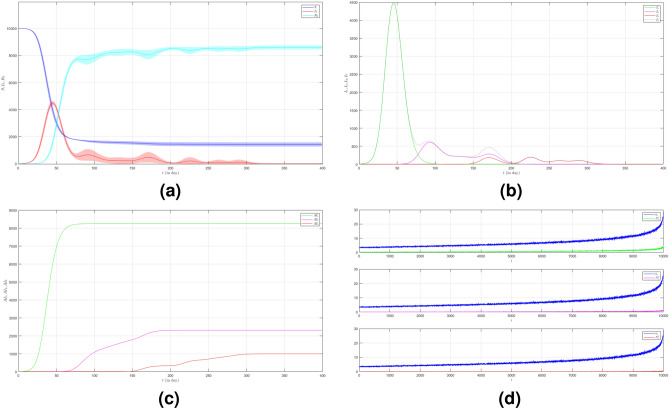
The dynamics of the epidemic process with three variants on a synthesized human interaction network for the first case of Scenario 1. (**a**) The time evolution of all states with $95\%$ confidence interval. The red lines indicate all infected nodes from all variants. (**b**) The average of 100 infection plots for each variant. The infection peaks of all variants are indicated by $I_{1_{46}}=4480$, $I_{2_{94}}=614$, and $I_{3_{227}}=197$ respectively. (**c**) The total number of infections for all variants are $AI^{T}_{1_{400}}=8250$, $AI^{T}_{2_{400}}=2305$, and $AI^{T}_{3_{400}}=992$. (**d**) Comparison between the number of infected neighbors and each node’s degree for all variants. The infection spread number are $\rho _{1}=0.241$, $\rho _{2}=0.109$, and $\rho _{3}=0.049$ respectively.

From the black histogram in Fig. 1, we can infer that there are fewer nodes with no degree. We call them isolated nodes. Almost all nodes in the network are connected. In addition, there are also many hubs in the network with degree 14–27. From the blue histogram in Fig. 1, we can infer that there are a lot of nodes with very high frequency of contact. Even, there are some nodes with contacts in the range 40–80. It makes sense if the disease spreads quickly as shown in Figure 2a. The figure is the time evolution of the number of individuals of all states (S, I, R) with $95\%$ confidence interval. We divide this scenario into four cases.

In the first case we set $\mu _{_{1 \rightarrow 2}}\sim \text {Bernoulli}(0.005), \; \mu _{_{2 \rightarrow 3}}\sim \text {Bernoulli}(0.005)$ and $\beta _{1}=\beta _{2}=\beta _{3}=0.01$. The emergence of variants causes multiple waves as shown in Fig. 2a as the red line. We can see the emergence of each variant and their time evolution in detail in Fig. 2b. The figure is the average plot of 100 samples. The infection peak of Variant 1 is 4480 nodes at the time $t=46$. The infection of Variant 2 is 614 nodes at the time $t=94$. The infection of Variant 3 is 197 at the time $t=227$. Variant 1 ends at time $t=118$. Variant 2 starts to spread at time $t=37$ and ends at time $t=227$. Variant 3 starts to spread at time $t=140$ and ends at time $t=337$. We can see the final value of the total number of infected individuals for each variant in Fig. 2c. From the total number of infected individuals of Variant 1 $AI^{T}_{1_{400}}=8250$ and the length of epidemic time of Variant 1 is $t_{e_{1}}=117$ days, we can find the infection spread number of Variant 1 $\rho _{1}=0.241$. For Variant 2 and Variant 3, we obtain $AI^{T}_{2_{400}}=2305$ with $t_{e_{2}}=190$ and $AI^{T}_{3_{400}}=992$ with $t_{e_{3}}=197$. Then, we have $\rho _{2}=0.109$ and $\rho _{3}=0.049$. We can compare the number of infected neighbors to the degree of each node in Fig. 2d. The higher the degree of a node the more neighbor will be infected.

**Figure 3:**
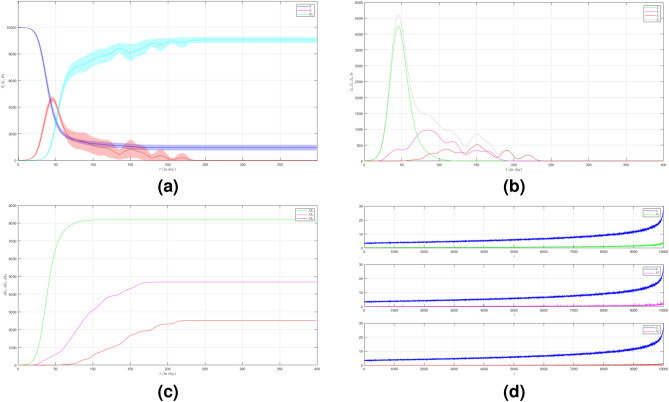
The dynamics of the epidemic process with three variants on a synthesized human interaction network for the second case of Scenario 1. (**a**) The time evolution of all states with $95\%$ confidence interval. The red lines indicate all infected nodes from all variants. (**b**) The average of 100 infection plots for each variant. The infection peaks of all variants are indicated by $I_{1_{46}}=4241$, $I_{2_{87}}=977$, and $I_{3_{152}}=519$ respectively. (**c**) The total number of infections for all variants are $AI^{T}_{1_{400}}=8181$, $AI^{T}_{2_{400}}=4675$, and $AI^{T}_{3_{400}}=2510$. (**d**) Comparison between the number of infected neighbors and each node’s degree for all variants. The infection spread number are $\rho _{1}=0.272$, $\rho _{2}=0.218$, and $\rho _{3}=0.122$ respectively.

In the second case we still set $\mu _{_{1 \rightarrow 2}}\sim \text {Bernoulli}(0.005), \; \mu _{_{2 \rightarrow 3}}\sim \text {Bernoulli}(0.005)$ but $\beta _{1}<\beta _{2}<\beta _{3}$. We choose $\beta _{1}=0.01, \; \beta _{2}=0.02$ and $\beta _{3}=0.03$ to show how different infection rates play roles in the epidemic process. The dynamic of the epidemic process in this case is shown in Fig. 3a. We can see that Variant 2 and Variant 3 emerge earlier as shown in Fig. 3b compared to Fig. 2b. The infection peak of Variant 1 is 4241 nodes at the time $t=46$. The infection peak of Variant 2 is 4675 nodes at the time $t=87$. The infection peak of Variant 3 is 2510 at the time $t=152$. Variant 1 ends at time $t=134$. Variant 2 starts to spread at time $t=15$ and ends at time $t=202$. Variant 3 starts to spread at time $t=51$ and ends at time $t=245$. The final value of the total number of infected individuals for each variant is shown in Fig. 2c. From the total number of infected individuals of Variant 1 $AI^{T}_{1_{400}}=8181$ and the length of epidemic time $t_{e_{1}}=133$ days, we can find the infection spread number of Variant 1 $\rho _{1}=0.272$. For Variant 2 and Variant 3, we obtain $AI^{T}_{2_{400}}=4675$ with $t_{e_{2}}=187$ and $AI^{T}_{3_{400}}=2510$ with $t_{e_{3}}=194$. We obtain $\rho _{2}=0.218$ and $\rho _{3}=0.122$. The increase in the infection rate for each variant can increase the total number of infected individuals and in turn, increase the infection spread number.

**Figure 4:**
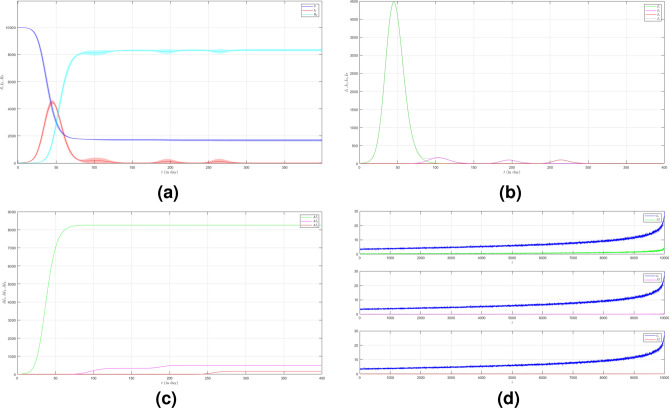
The dynamics of the epidemic process with three variants on a synthesized human interaction network for the third case of Scenario 1. (**a**) The time evolution of all states with $95\%$ confidence interval. The red lines indicate all infected nodes from all variants. (**b**) The average of 100 infection plots for each variant. The infection peaks of all variants are indicated by $I_{1_{46}}=4482$, $I_{2_{104}}=158$, and $I_{3_{265}}=99$ respectively. (**c**) The total number of infections for all variants are $AI^{T}_{1_{400}}=8256$, $AI^{T}_{2_{400}}=496$, and $AI^{T}_{3_{400}}=166$. (**d**) Comparison between the number of infected neighbors and each node’s degree for all variants. The infection spread number are $\rho _{1}=0.243$, $\rho _{2}=0.022$, and $\rho _{3}=0.003$ respectively.

Now we turn to the third case where we set $\mu _{_{1 \rightarrow 2}}\sim \text {Bernoulli}(0.001), \; \mu _{_{2 \rightarrow 3}}\sim \text {Bernoulli}(0.001)$ and $\beta _{1}=\beta _{2}=\beta _{3}=0.01$. Here we decrease the probability of the emergence of new variants. The dynamic of the epidemic process in this case is shown in Fig. 4a. The infected individuals are dominated by Variant 1 as shown in Fig. 4b. The infection peak of Variant 1 is 4482 nodes at the time $t=46$. The infection peak of Variant 2 is 158 nodes at the time $t=104$. The infection peak of Variant 3 is 99 at the time $t=265$. Variant 1 ends at time $t=119$. Variant 2 starts to spread at time $t=65$ and ends at time $t=240$. Variant 3 starts to spread at time $t=232$ and ends at time $t=306$. The final value of the total number of infected individuals for Variant 2 and Variant 3 are much smaller than the total number of infected individuals for Variant 1 as shown in Fig. 4c. The total number of infected individuals of Variant 1 $AI^{T}_{1_{400}}=8256$ and the epidemic duration $t_{e_{1}}=118$ days give the infection spread number of Variant 1 $\rho _{1}=0.243$. For Variant 2 and Variant 3, we obtain $AI^{T}_{2_{400}}=496$ with $t_{e_{2}}=175$ and $AI^{T}_{3_{400}}=166$ with $t_{e_{3}}=74$. We have $\rho _{2}=0.022$ and $\rho _{3}=0.003$. The smaller probability of the emergence of new variants can reduce the total number of infected individuals of new variants.

**Figure 5:**
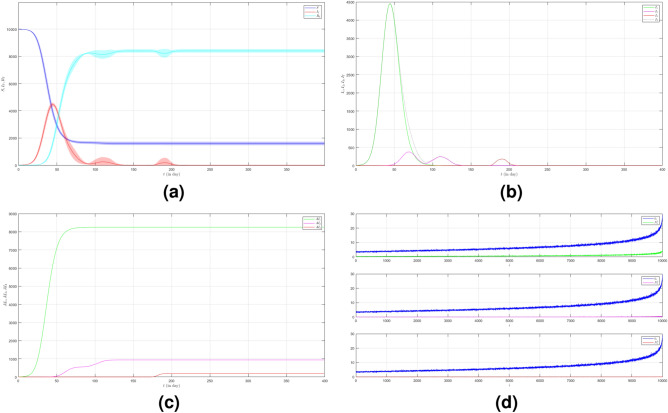
The dynamics of the epidemic process with three variants on a synthesized human interaction network for the fourth case of Scenario 1. (**a**) The time evolution of all states with $95\%$ confidence interval. The red lines indicate all infected nodes from all variants. (**b**) The average of 100 infection plots for each variant. The infection peaks of all variants are indicated by $I_{1_{45}}=4448$, $I_{2_{70}}=376$, and $I_{3_{192}}=181$ respectively. (**c**) The total number of infections for all variants are $AI^{T}_{1_{400}}=8249$, $AI^{T}_{2_{400}}=939$, and $AI^{T}_{3_{400}}=195$. (**d**) Comparison between the number of infected neighbors and each node’s degree for all variants. The infection spread numbers are $\rho _{1}=0.239$, $\rho _{2}=0.027$, and $\rho _{3}=0.002$ respectively.

To see how different infection rates from each variant play roles in a condition when the probability of the emergence of new variants is smaller, we set the fourth case where $\mu _{_{1 \rightarrow 2}}\sim \text {Bernoulli}(0.001), \; \mu _{_{2 \rightarrow 3}}\sim \text {Bernoulli}(0.001)$ and $\beta _{1}<\beta _{2}<\beta _{3}$. We still choose $\beta _{1}=0.01, \; \beta _{2}=0.02$ and $\beta _{3}=0.03$. The dynamic of the epidemic process in this case is shown in Fig. 5a. The infected individuals are still dominated by Variant 1 as shown in Fig. 5b. The infection peak of Variant 1 is 4448 nodes at the time $t=45$. The infection peak of Variant 2 is 376 nodes at the time $t=70$. The infection peak of Variant 3 is 181 at the time $t=192$. Variant 2 starts to spread at time $t=30$ and ends at time $t=147$. Variant 1 ends at time $t=117$. Variant 3 starts to spread at time $t=174$ and ends at time $t=217$. The smaller probability of the emergence of new variants causes the final values of the total number of infected individuals for Variant 2 and Variant 3 to be smaller than the total number of infected individuals for Variant 1 as shown in Fig. 5c. The total number of infected individuals of Variant 1 $AI^{T}_{1_{400}}=8249$ and the epidemic duration $t_{e_{1}}=116$ days give the infection spread number of Variant 1 $\rho _{1}=0.239$. For Variant 2 and Variant 3, we obtain $AI^{T}_{2_{400}}=939$ with $t_{e_{2}}=117$ and $AI^{T}_{3_{400}}=195$ with $t_{e_{3}}=43$. We have $\rho _{2}=0.027$ and $\rho _{3}=0.002$. Although a smaller probability of the emergence of new variants can reduce the total number of infected individuals of new variants, the higher infection rates still give a significant increase in the total number of infected individuals for each variant.

### Scenario 2

**Figure 6:**
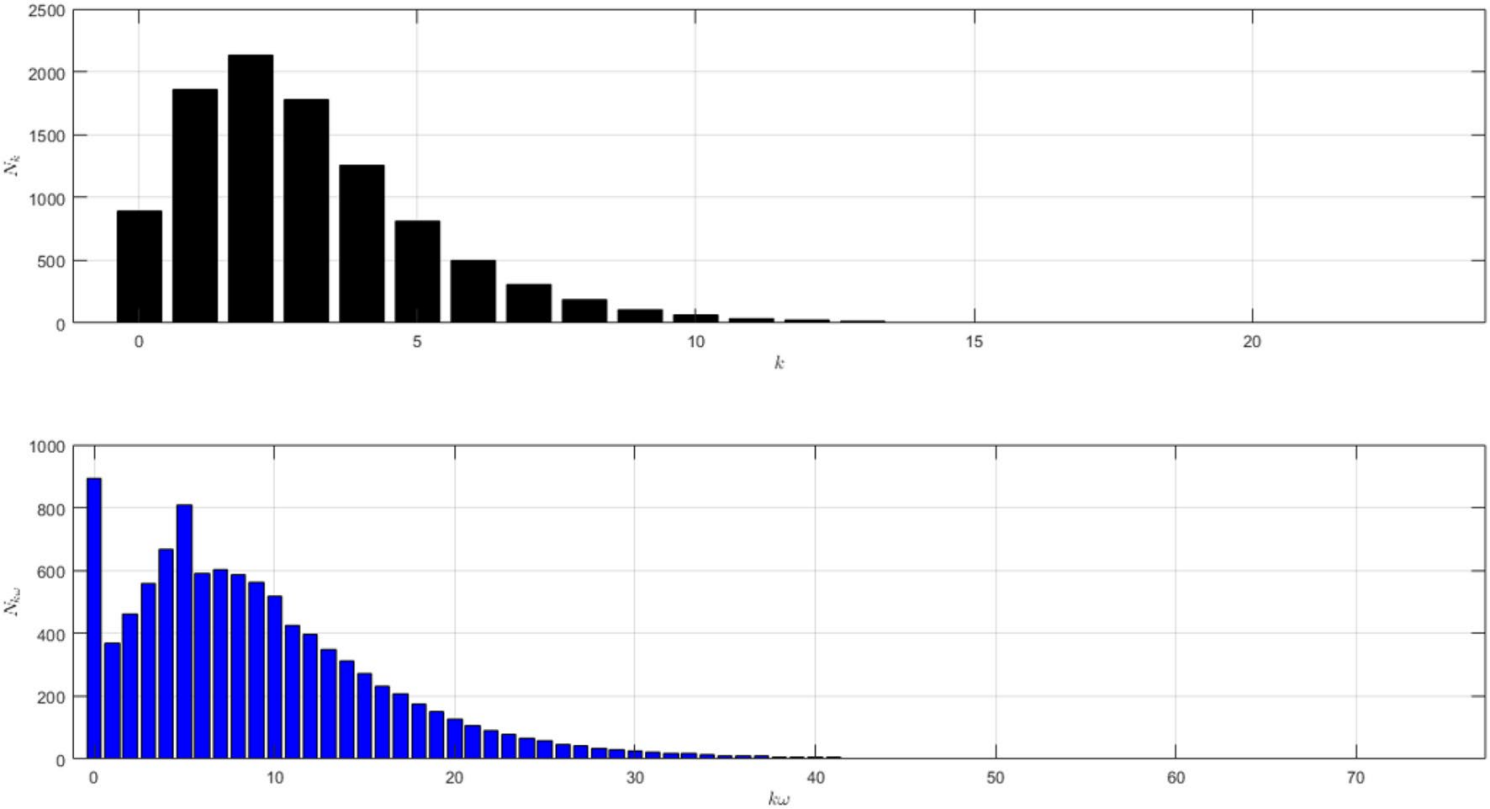
The network structure of Scenario 2. The black histogram shows the network’s degree distribution with ${\bar{k}}=3$ and $\omega =5$. The highest degree is 13. The network is dominated by the nodes with degree 2. The blue histogram shows the distribution of the total number of contacts. More than half of the population have a contact frequency of $10-40$.

The network structure of Scenario 2 where ${\bar{k}}=3$ and $\omega =5$ is shown in Fig. 6. In this scenario, we contain the spread of infection in the network by implementing self-quarantine without physical distancing. We reduce individual connectivity without reducing their contact frequency.

**Figure 7:**
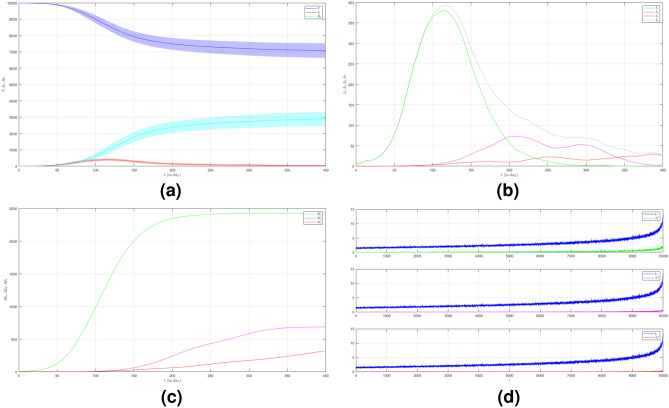
The dynamics of the epidemic process with three variants on a synthesized human interaction network for the first case of Scenario 2. (**a**) The time evolution of all states with $95\%$ confidence interval. The red lines indicate all infected nodes from all variants. (**b**) The average of 100 infection plots for each variant. The infection peaks of all variants are indicated by $I_{1_{115}}=381$, $I_{2_{212}}=74$, and $I_{3_{384}}=30$ respectively. (**c**) The total number of infections for all variants are $AI^{T}_{1_{400}}=2426$, $AI^{T}_{2_{400}}=688$, and $AI^{T}_{3_{400}}=319$. (**d**) Comparison between the number of infected neighbors and each node’s degree for all variants. The infection spread numbers are $\rho _{1}=0.198$, $\rho _{2}=0.058$, and $\rho _{3}=0.025$ respectively.

The black histogram in Fig. 1 shows that there is no hub in this network. From the blue histogram in Fig. 1, there are fewer nodes with contacts in the range $30-40$ which is the high contacts in the network. The dynamic of the epidemic process is shown in Fig. 7a. The figure is the time evolution of the number of individuals of all states (S, I, R) with $95\%$ confidence interval. Here, we still divide this scenario into four cases.

In the first case of Scenario 2, we set $\mu _{_{1 \rightarrow 2}}\sim \text {Bernoulli}(0.005), \; \mu _{_{2 \rightarrow 3}}\sim \text {Bernoulli}(0.005)$ and $\beta _{1}=\beta _{2}=\beta _{3}=0.01$. The number of infected individuals from all variants indicated by the red line is shown in Fig. 7a. The emergence of new variants is shown in Fig. 7b. The figure is the average plot of 100 samples. The infection peak of Variant 1 is 381 nodes at the time $t=115$. The infection of Variant 2 is 74 nodes at the time $t=212$. The infection of Variant 3 is 30 at the time $t=384$. Variant 1 ends at time $t=328$. Variant 2 starts to spread at time $t=64$ and it does not end even until the last time. Variant 3 starts to spread at time $t=83$ and it does not end either. We can see the final value of the total number of infected individuals for each variant in Fig. 7c. From the total number of infected individuals of Variant 1 $AI^{T}_{1_{400}}=2426$ and the length of epidemic time of Variant 1 is $t_{e_{1}}=327$ days, we can find the infection spread number of Variant 1 $\rho _{1}=0.198$. For Variant 2 and Variant 3, we obtain $AI^{T}_{2_{400}}=688$ with $t_{e_{2}}=336$ and $AI^{T}_{3_{400}}=319$ with $t_{e_{3}}=317$. Then, we have $\rho _{2}=0.058$ and $\rho _{3}=0.025$. In this case, reducing the connectivity of each individual by reducing the average degree prolongs the length of the epidemic time of each variant. We can compare the number of infected neighbors to the degree of each node in Fig. 7d. The higher the degree of a node the more neighbor will be infected.

**Figure 8:**
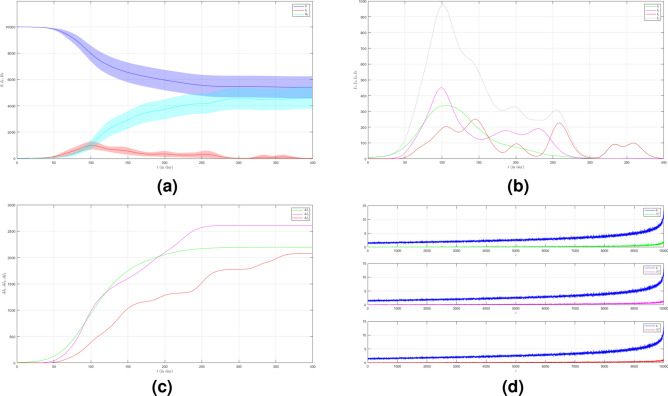
The dynamics of the epidemic process with three variants on a synthesized human interaction network for the second case of Scenario 2. (**a**) The time evolution of all states with $95\%$ confidence interval. The red lines indicate all infected nodes from all variants. (**b**) The average of 100 infection plots for each variant. The infection peaks of all variants are indicated by $I_{1_{105}}=337$, $I_{2_{101}}=451$, and $I_{3_{145}}=251$ respectively. (**c**) The total number of infections for all variants are $AI^{T}_{1_{400}}=2195$, $AI^{T}_{2_{400}}=2610$, and $AI^{T}_{3_{400}}=2078$. (**d**) Comparison between the number of infected neighbors and each node’s degree for all variants. The infection spread numbers are $\rho _{1}=0.182$, $\rho _{2}=0.181$, and $\rho _{3}=0.186$ respectively.

In the second case of Scenario 2, we still set $\mu _{_{1 \rightarrow 2}}\sim \text {Bernoulli}(0.005), \; \mu _{_{2 \rightarrow 3}}\sim \text {Bernoulli}(0.005)$ but $\beta _{1}<\beta _{2}<\beta _{3}$. We choose $\beta _{1}=0.01, \; \beta _{2}=0.02$ and $\beta _{3}=0.03$ to show how different infection rates play roles in the epidemic process when the average degree is reduced. The dynamic of the epidemic process in this case is shown in Fig. 8a. There is an increase in the number of infections due to Variant 2 and Variant 3 as shown in Fig. 8b compared to Fig. 7b. The emergence of new variants is earlier than the emergence of new variants in the first case. The infection peak of Variant 1 is 337 nodes at the time $t=105$. The infection peak of Variant 2 is 451 nodes at the time $t=101$. The infection peak of Variant 3 is 251 at the time $t=145$. Variant 1 ends at time $t=332$. Variant 2 starts to spread at time $t=29$ and ends at time $t=306$. Variant 3 starts to spread at time $t=42$ and does not end until the last time. The final value of the total number of infected individuals for each variant is shown in Fig. 8c. From the total number of infected individuals of Variant 1 $AI^{T}_{1_{400}}=2195$ and the length of epidemic time $t_{e_{1}}=331$ days, we can find the infection spread number of Variant 1 $\rho _{1}=0.182$. For Variant 2 and Variant 3, we obtain $AI^{T}_{2_{400}}=2610$ with $t_{e_{2}}=277$ and $AI^{T}_{3_{400}}=2078$ with $t_{e_{3}}=358$. We obtain $\rho _{2}=0.181$ and $\rho _{3}=0.186$.

**Figure 9:**
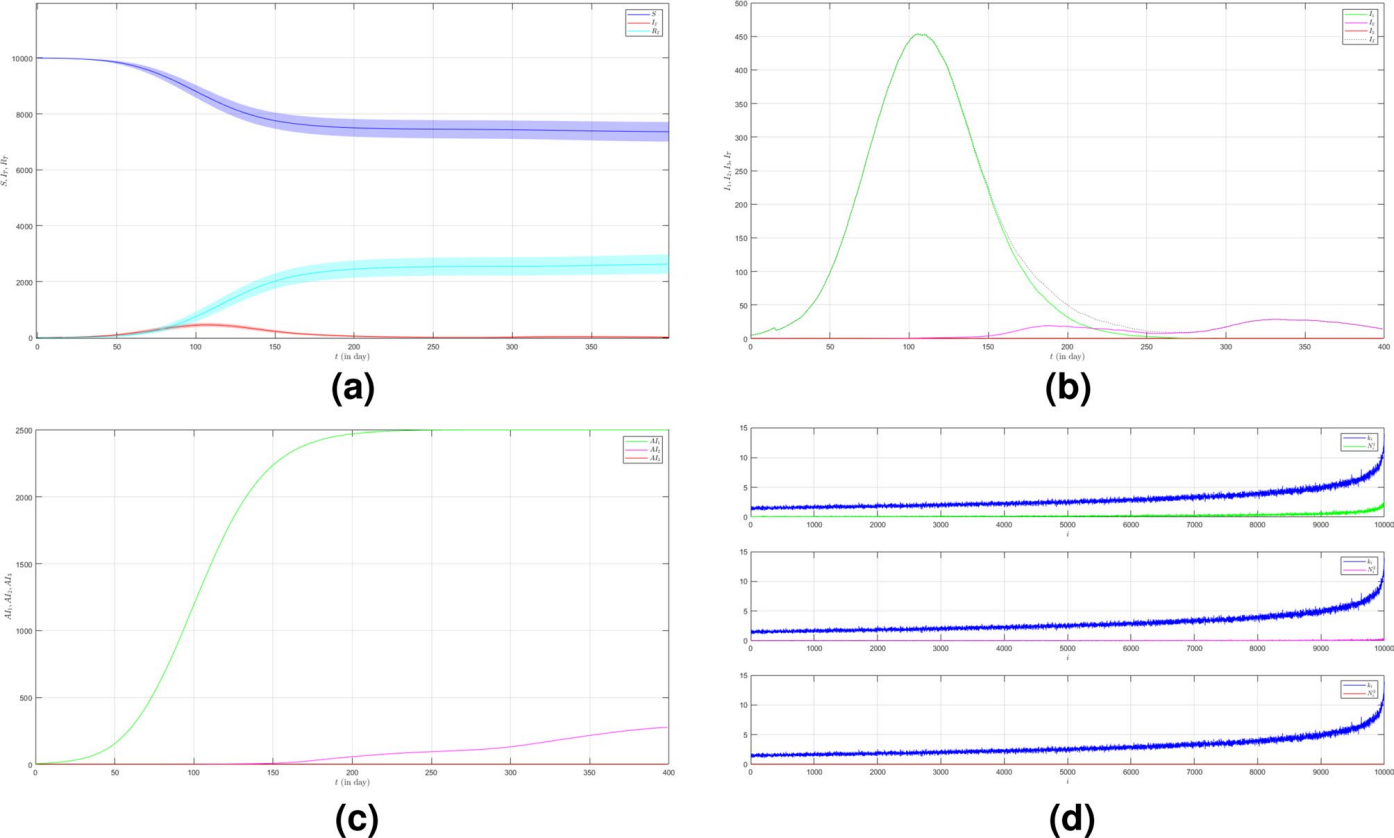
The dynamics of the epidemic process with three variants on a synthesized human interaction network for the third case of Scenario 2. (**a**) The time evolution of all states with $95\%$ confidence interval. The red lines indicate all infected nodes from all variants. (**b**) The average of 100 infection plots for each variant. The infection peaks of all variants are indicated by $I_{1_{106}}=454$, $I_{2_{334}}=29$, and $I_{3_{139}}=1$ respectively. (**c**) The total number of infections for all variants are $AI^{T}_{1_{400}}=2498$, $AI^{T}_{2_{400}}=277$, and $AI^{T}_{3_{400}}=1$. (**d**) Comparison between the number of infected neighbors and each node’s degree for all variants. The infection spread numbers are $\rho _{1}=0.168$, $\rho _{2}=0.019$, and $\rho _{3}=0$ respectively.

Now we set the third case of Scenario 2 where $\mu _{_{1 \rightarrow 2}}\sim \text {Bernoulli}(0.001), \; \mu _{_{2 \rightarrow 3}}\sim \text {Bernoulli}(0.001)$ and $\beta _{1}=\beta _{2}=\beta _{3}=0.01$. The dynamic of the epidemic process in this case is shown in Fig. 9a. The infection of Variant 2 and Variant 3 is smaller than the two previous cases as shown in Fig. 9b. The infection peak of Variant 1 is 454 nodes at the time $t=106$. The infection peak of Variant 2 is 29 nodes at the time $t=334$. There is no spread of Variant 3. Variant 1 ends at time $t=270$. Variant 2 starts to spread at time $t=115$ and does not end until the last time. The final value of the total number of infected individuals for Variant 2 and Variant 3 are much smaller than the total number of infected individuals for Variant 1 as shown in Fig. 9c. The total number of infected individuals of Variant 1 $AI^{T}_{1_{400}}=2498$ and the epidemic duration $t_{e_{1}}=269$ days give the infection spread number of Variant 1 $\rho _{1}=0.168$. For Variant 2 and Variant 3, we obtain $AI^{T}_{2_{400}}=277$ with $t_{e_{2}}=285$ and $AI^{T}_{3_{400}}=1$. We have $\rho _{2}=0.019$ and $\rho _{3}=0$.

**Figure 10:**
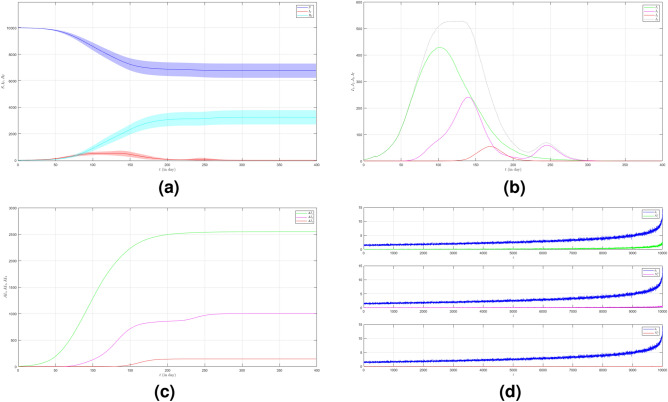
The dynamics of the epidemic process with three variants on a synthesized human interaction network for the fourth case of Scenario 2. (**a**) The time evolution of all states with $95\%$ confidence interval. The red lines indicate all infected nodes from all variants. (**b**) The average of 100 infection plots for each variant. The infection peaks of all variants are indicated by $I_{1_{102}}=429$, $I_{2_{140}}=241$, and $I_{3_{169}}=56$ respectively. (**c**) The total number of infections for all variants are $AI^{T}_{1_{400}}=2547$, $AI^{T}_{2_{400}}=1000$, and $AI^{T}_{3_{400}}=143$. (**d**) Comparison between the number of infected neighbors and each node’s degree for all variants. The infection spread numbers are $\rho _{1}=0.192$, $\rho _{2}=0.061$, and $\rho _{3}=0.003$ respectively.

To see how different infection rates from each variant play roles in a condition when the probability of the emergence of new variants is smaller, we set the fourth case of Scenario 2 where $\mu _{_{1 \rightarrow 2}}\sim \text {Bernoulli}(0.001), \; \mu _{_{2 \rightarrow 3}}\sim \text {Bernoulli}(0.001)$ and $\beta _{1}<\beta _{2}<\beta _{3}$. We still choose $\beta _{1}=0.01, \; \beta _{2}=0.02$ and $\beta _{3}=0.03$. The dynamic of the epidemic process in this case is shown in Fig. 10a. The time evolution of infected individuals for each variant is shown in Fig. 10b. The infection peak of Variant 1 is 429 nodes at the time $t=102$. The infection peak of Variant 2 is 241 nodes at the time $t=140$. The infection peak of Variant 3 is 56 at the time $t=169$. Variant 1 ends at time $t=303$. Variant 2 starts to spread at time $t=59$ and ends at time $t=303$. Variant 3 starts to spread at time $t=125$ and ends at time $t=226$. The total number of infected individuals for each variant is shown in Fig. 10c. The total number of infected individuals of Variant 1 $AI^{T}_{1_{400}}=2547$ and the epidemic duration $t_{e_{1}}=302$ days give the infection spread number of Variant 1 $\rho _{1}=0.192$. For Variant 2 and Variant 3, we obtain $AI^{T}_{2_{400}}=1000$ with $t_{e_{2}}=244$ and $AI^{T}_{3_{400}}=143$ with $t_{e_{3}}=101$. We have $\rho _{2}=0.061$ and $\rho _{3}=0.003$.

### Scenario 3

**Figure 11:**
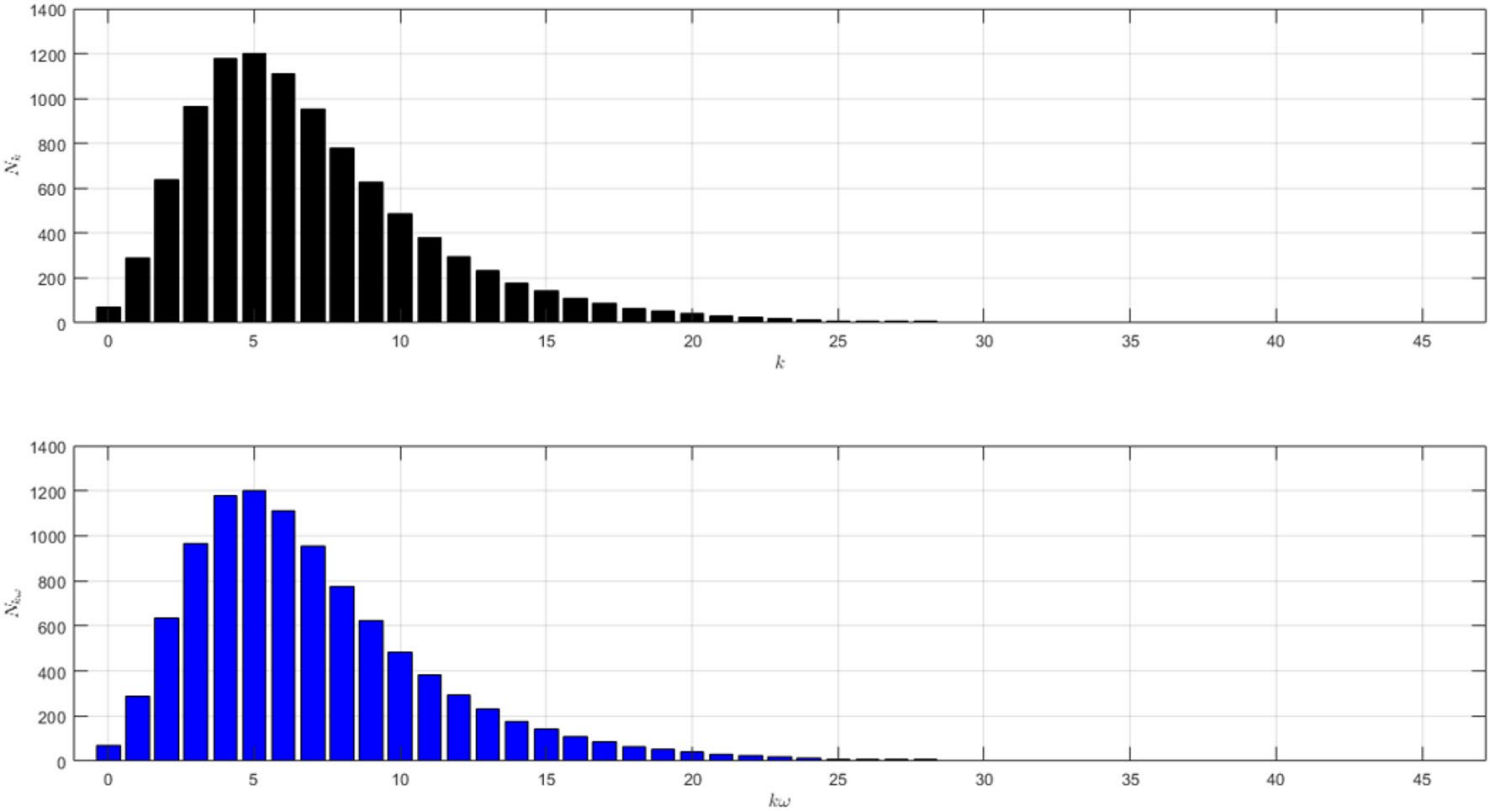
The network structure of Scenario 3. The black histogram shows the network’s degree distribution with ${\bar{k}}=7$ and $\omega =1$. It is the same distribution as the blue histogram because the contact frequency is 1 for each link in the network. There are many hubs with a contact frequency of $14-28$.

We assume that the network structure of Scenario 3 where ${\bar{k}}=7$ and $\omega =1$ is the epidemic process in the network with measured physical distancing but no self-quarantine as shown in Fig. 11. In this scenario, we contain the spread of infection in the network by implementing physical distancing without self-quarantine. We reduce the individual frequency of contact without reducing the connectivity. Here, we still divide this scenario into four cases.

**Figure 12:**
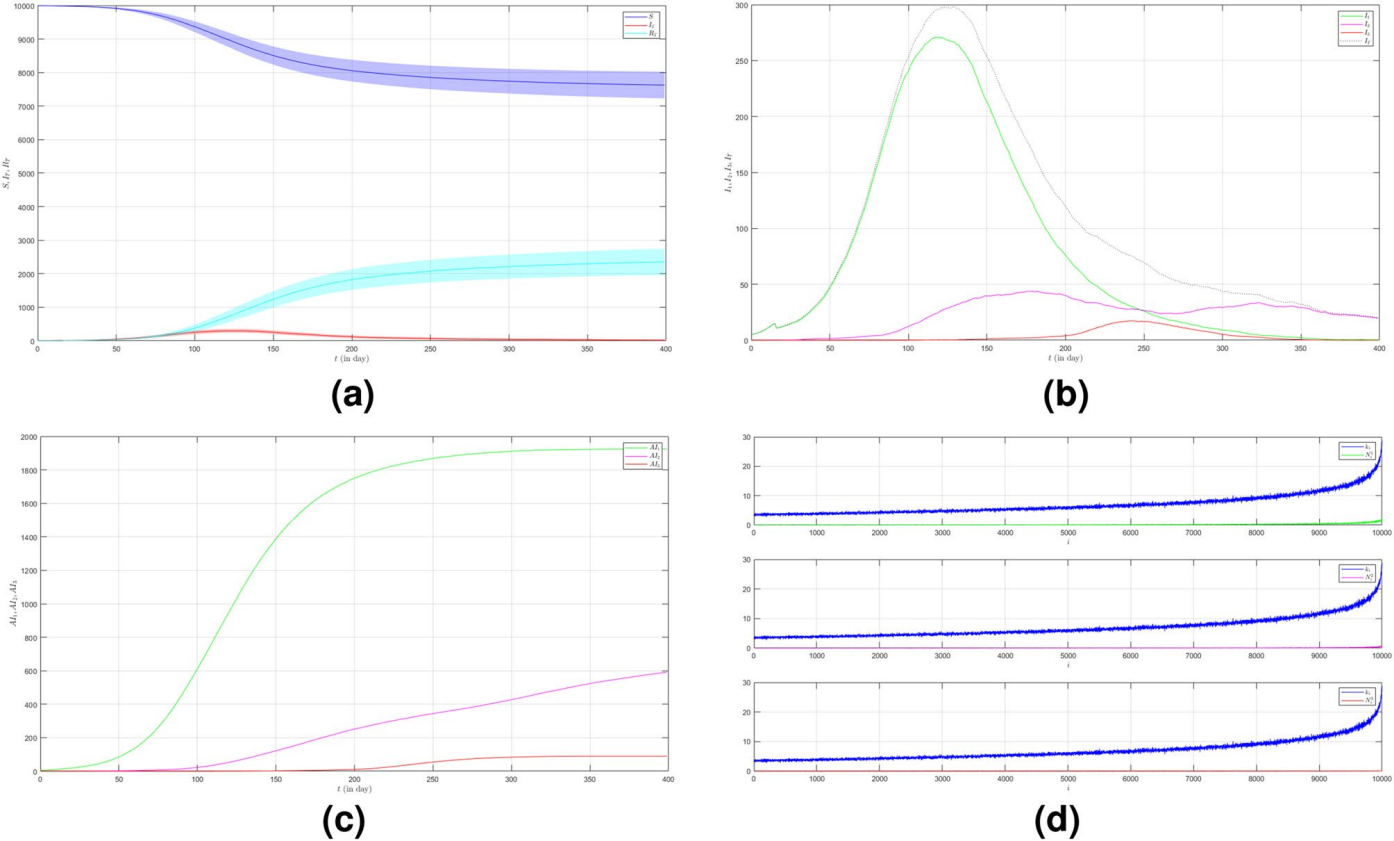
The dynamics of the epidemic process with three variants on a synthesized human interaction network for the first case of Scenario 3. (**a**) The time evolution of all states with $95\%$ confidence interval. The red lines indicate all infected nodes from all variants. (**b**) The average of 100 infection plots for each variant. The infection peaks of all variants are indicated by $I_{1_{122}}=271$, $I_{2_{177}}=44$, and $I_{3_{244}}=18$ respectively. (**c**) The total number of infections for all variants are $AI^{T}_{1_{400}}=1925$, $AI^{T}_{2_{400}}=592$, and $AI^{T}_{3_{400}}=90$. (**d**) Comparison between the number of infected neighbors and each node’s degree for all variants. The infection spread numbers are $\rho _{1}=0.175$, $\rho _{2}=0.054$, and $\rho _{3}=0.004$ respectively.

The black histogram in Fig. 11 is the same as the blue histogram. There are quite a lot of individuals who are hubs and have frequency contact of $14-28$. The dynamic of the epidemic process is shown in Fig. 12a. The figure is the time evolution of the number of individuals of all states (S,I, R) with $95\%$ confidence interval.

In the first case of Scenario 3, we set $\mu _{_{1 \rightarrow 2}}\sim \text {Bernoulli}(0.005), \; \mu _{_{2 \rightarrow 3}}\sim \text {Bernoulli}(0.005)$ and $\beta _{1}=\beta _{2}=\beta _{3}=0.01$. The number of infected individuals from all variants indicated by the red line is shown in Fig. 12a. Although the infected individuals are still dominated by the infection of Variant 1, the total number of infected individuals is much smaller than in all previous cases we obtained above. The emergence of new variants is shown in Fig. 12b. The figure is the average plot of 100 samples. We can see that the time evolution of infection of Variant 2 has no end. The infection peak of Variant 1 is 271 nodes at the time $t=122$. The infection of Variant 2 is 44 nodes at the time $t=177$. The infection of Variant 3 is 18 at the time $t=244$. Variant 1 ends at time $t=364$. Variant 2 starts to spread at time $t=38$ and it does not end even until the last time. Variant 3 starts to spread at time $t=148$ and it ends at time $t=343$. We can see the final value of the total number of infected individuals for each variant in Fig. 12c. From the total number of infected individuals of Variant 1 $AI^{T}_{1_{400}}=1925$ and the length of epidemic time of Variant 1 is $t_{e_{1}}=363$ days, we can find the infection spread number of Variant 1 $\rho _{1}=0.175$. For Variant 2 and Variant 3, we obtain $AI^{T}_{2_{400}}=592$ with $t_{e_{2}}=362$ and $AI^{T}_{3_{400}}=90$ with $t_{e_{3}}=195$. Then, we have $\rho _{2}=0.054$ and $\rho _{3}=0.004$. In this case, reducing the frequency of contact also prolongs the length of the epidemic time of each variant. We can compare the number of infected neighbors to the degree of each node in Fig. 12d.

**Figure 13:**
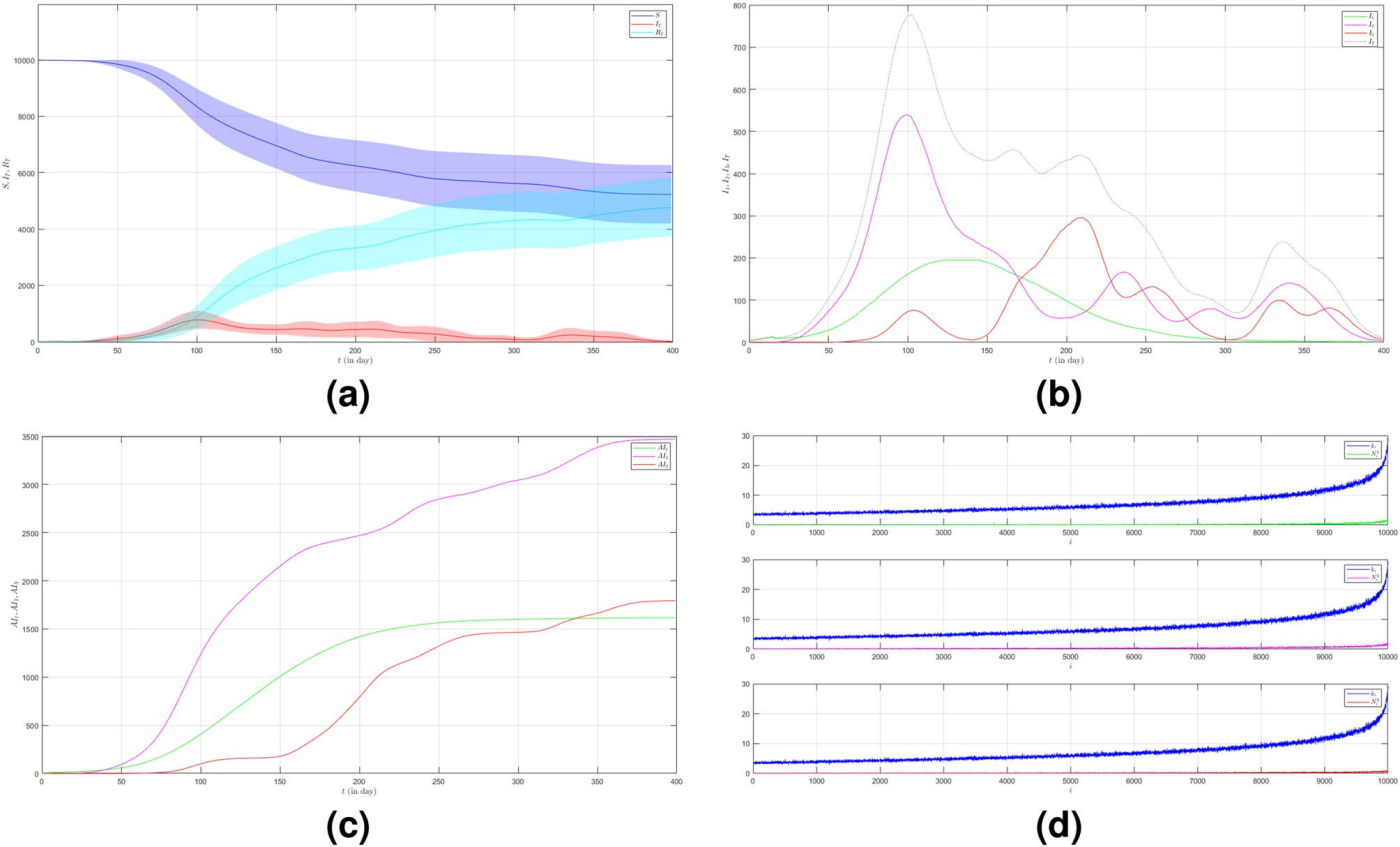
The dynamics of the epidemic process with three variants on a synthesized human interaction network for the second case of Scenario 3. (**a**) The time evolution of all states with $95\%$ confidence interval. The red lines indicate all infected nodes from all variants. (**b**) The average of 100 infection plots for each variant. The infection peaks of all variants are indicated by $I_{1_{139}}=196$, $I_{2_{100}}=540$, and $I_{3_{211}}=297$ respectively. (**c**) The total number of infections for all variants are $AI^{T}_{1_{400}}=1617$, $AI^{T}_{2_{400}}=3472$, and $AI^{T}_{3_{400}}=1794$. (**d**) Comparison between the number of infected neighbors and each node’s degree for all variants. The infection spread number of all variants are $\rho _{1}=0.161$, $\rho _{2}=0.331$, and $\rho _{3}=0.153$ respectively.

In the second case of Scenario 3, we still set $\mu _{_{1 \rightarrow 2}}\sim \text {Bernoulli}(0.005), \; \mu _{_{2 \rightarrow 3}}\sim \text {Bernoulli}(0.005)$ but $\beta _{1}<\beta _{2}<\beta _{3}$. We still choose $\beta _{1}=0.01, \; \beta _{2}=0.02$ and $\beta _{3}=0.03$ to show how different infection rates play roles in the epidemic process when the frequency of contact is reduced. The dynamic of the epidemic process in this case is shown in Fig. 13a. There is an increase in the number of infections due to Variant 2 and Variant 3 as shown in Fig. 13b compared to Fig. 12b. The emergence of new variants is earlier than the emergence of new variants in the first case. The infection peak of Variant 1 is 196 nodes at the time $t=139$. The infection peak of Variant 2 is 540 nodes at the time $t=100$. The infection peak of Variant 3 is 297 at the time $t=211$. Variant 1 spreads until the last time. Variant 2 starts to spread at time $t=19$ and has no end. Variant 3 starts to spread at time $t=59$ and has no end either. The final value of the total number of infected individuals for each variant is shown in Fig. 13c. From the total number of infected individuals of Variant 1 $AI^{T}_{1_{400}}=1617$ and the length of epidemic time $t_{e_{1}}=399$ days, we can find the infection spread number of Variant 1 $\rho _{1}=0.161$. For Variant 2 and Variant 3, we obtain $AI^{T}_{2_{400}}=3472$ with $t_{e_{2}}=381$ and $AI^{T}_{3_{400}}=1794$ with $t_{e_{3}}=341$. We obtain $\rho _{2}=0.331$ and $\rho _{3}=0.153$. In this case, if the infection rate of the new variant is larger than the previous one, we can see that not only does the total number of infected individuals increase but also the length of the epidemic time is longer.

**Figure 14:**
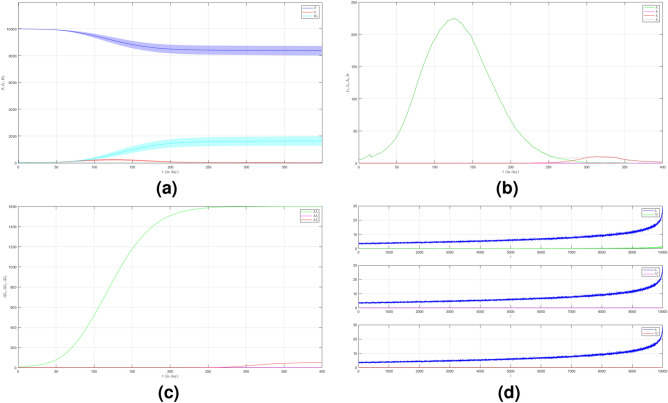
The dynamics of the epidemic process with three variants on a synthesized human interaction network for the third case of Scenario 3. (**a**) The time evolution of all states with $95\%$ confidence interval. The red lines indicate all infected nodes from all variants. (**b**) The average of 100 infection plots for each variant. The infection peaks of all variants are indicated by $I_{1_{126}}=226$, $I_{2_{127}}=1$, and $I_{3_{314}}=10$ respectively. (**c**) The total number of infections for all variants are $AI^{T}_{1_{400}}=1599$, $AI^{T}_{2_{400}}=1$, and $AI^{T}_{3_{400}}=51$. (**d**) Comparison between the number of infected neighbors and each node’s degree for all variants. The infection spread numbers are $\rho _{1}=0.121$, $\rho _{2}=0$, and $\rho _{3}=0.002$ respectively.

Now we set the third case of Scenario 3 where $\mu _{_{1 \rightarrow 2}}\sim \text {Bernoulli}(0.001), \; \mu _{_{2 \rightarrow 3}}\sim \text {Bernoulli}(0.001)$ and $\beta _{1}=\beta _{2}=\beta _{3}=0.01$. In this case, we want to see what happens if we reduce the probability of the emergence of new variants. The dynamic of the epidemic process in this case is shown in Fig. 14a. We can see that the time evolution of total infections indicated by the red line increases very slowly and levels off for quite a long time, then decreases until the end. The infection number of Variant 2 and Variant 3 is much smaller as shown in Fig. 14b. The infection peak of Variant 1 is 226 nodes at the time $t=126$. The infection peak of Variant 2 is 1 nodes at the time $t=127$. It means there is only one infection of Variant 2. Thus, there is no spread of Variant 2. The infection peak of Variant 3 is 10 nodes at the time $t=314$. Variant 1 ends at time $t=304$. Variant 3 starts to spread at time $t=259$ and does not end until the last time. The final value of the total number of infected individuals for Variant 2 and Variant 3 are much smaller than the total number of infected individuals for Variant 1 as shown in Fig. 14c. The total number of infected individuals of Variant 1 $AI^{T}_{1_{400}}=1599$ and the epidemic duration $t_{e_{1}}=303$ days give the infection spread number of Variant 1 $\rho _{1}=0.0121$. Since there is no spread of Variant 2, we write $\rho _{2}=0$. For Variant 3, we obtain $AI^{T}_{2_{400}}=51$ with $t_{e_{2}}=141$ and then $\rho _{3}=0.002$.

**Figure 15:**
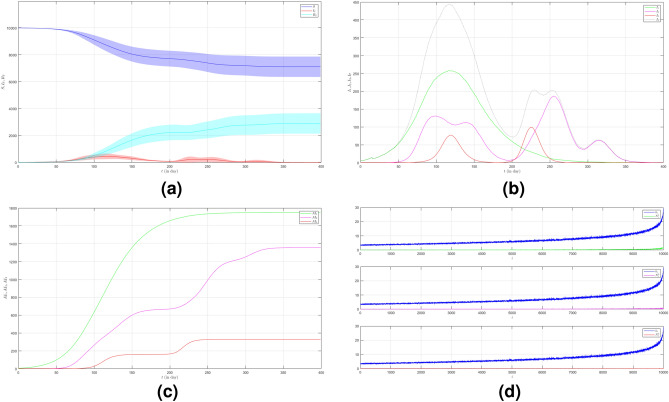
The dynamics of the epidemic process with three variants on a synthesized human interaction network for the fourth case of Scenario 3. (**a**) The time evolution of all states with $95\%$ confidence interval. The red lines indicate all infected nodes from all variants. (**b**) The average of 100 infection plots for each variant. The infection peaks of all variants are indicated by $I_{1_{118}}=258$, $I_{2_{256}}=187$, and $I_{3_{226}}=100$ respectively. (**c**) The total number of infections for all variants are $AI^{T}_{1_{400}}=1751$, $AI^{T}_{2_{400}}=1357$, and $AI^{T}_{3_{400}}=328$. (**d**) Comparison between the number of infected neighbors and each node’s degree for all variants. The infection spread numbers are $\rho _{1}=0.141$, $\rho _{2}=0.111$, and $\rho _{3}=0.008$ respectively.

To see how different infection rates from each variant play roles in a condition when the probability of the emergence of new variants is smaller and the frequency of contact is reduced, we set the fourth case of Scenario 3 where $\mu _{_{1 \rightarrow 2}}\sim \text {Bernoulli}(0.001), \; \mu _{_{2 \rightarrow 3}}\sim \text {Bernoulli}(0.001)$ and $\beta _{1}<\beta _{2}<\beta _{3}$. We still choose $\beta _{1}=0.01, \; \beta _{2}=0.02$ and $\beta _{3}=0.03$. The dynamic of the epidemic process in this case is shown in Fig. 15a. The time evolution of infected individuals for each variant is shown in Fig. 15b. The infection peak of Variant 1 is 258 nodes at the time $t=118$. The infection peak of Variant 2 is 187 nodes at the time $t=256$. The infection peak of Variant 3 is 100 at the time $t=226$. Variant 1 ends at time $t=323$. Variant 2 starts to spread at time $t=46$ and ends at time $t=375$. Variant 3 starts to spread at time $t=73$ and ends at time $t=173$. The total number of infected individuals for each variant is shown in Fig. 15c. The total number of infected individuals of Variant 1 $AI^{T}_{1_{400}}=1751$ and the epidemic duration $t_{e_{1}}=322$ days give the infection spread number of Variant 1 $\rho _{1}=0.141$. For Variant 2 and Variant 3, we obtain $AI^{T}_{2_{400}}=1357$ with $t_{e_{2}}=329$ and $AI^{T}_{3_{400}}=328$ with $t_{e_{3}}=100$. We have $\rho _{2}=0.111$ and $\rho _{3}=0.008$.

### Scenario 4

**Figure 16:**
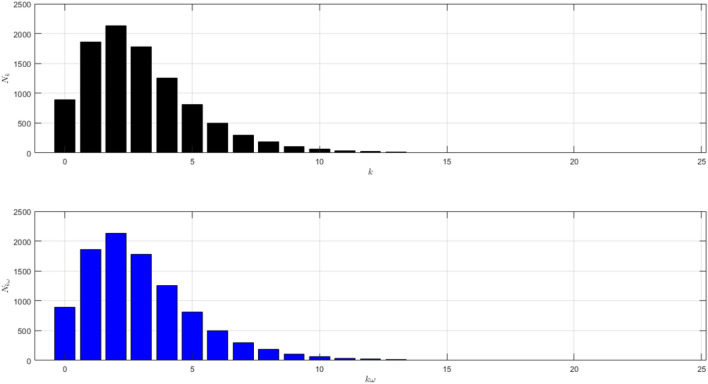
The network structure of Scenario 4. The black histogram shows the network’s degree distribution with ${\bar{k}}=3$ and $\omega =1$. The black histogram is the same as the blue one. There is no hub in this network.

We assume that the network structure of Scenario 4 where ${\bar{k}}=3$ and $\omega =1$ is the epidemic process in the network with measured physical distancing and self-quarantine as shown in Fig. 16. In this scenario, we contain the spread of infection in the network by implementing both self-quarantine and physical distancing. We reduce both the connectivity and the individual frequency of contact.

**Figure 17:**
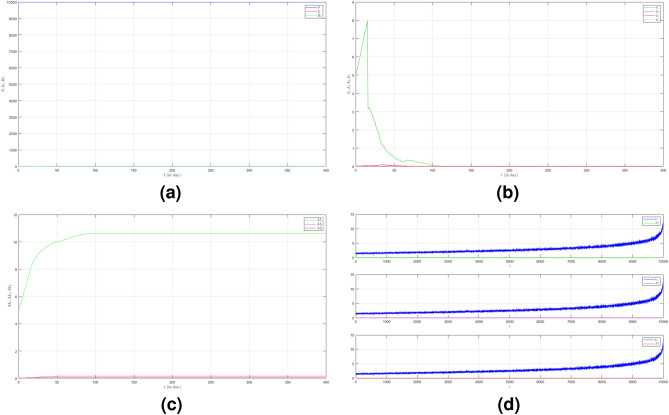
The dynamics of the epidemic process with three variants on a synthesized human interaction network for the first case of Scenario 4. (**a**) The time evolution of all states with $95\%$ confidence interval. The red lines indicate all infected nodes from all variants. (**b**) The average of 100 infection plots for each variant. The infection peaks of Variant 1 and Variant 2 are indicated by $I_{1_{16}}=8$ and $I_{2_{33}}=1$. (**c**) The total number of infections for all variants are $AI^{T}_{1_{400}}=11$, $AI^{T}_{2_{400}}=1$, and $AI^{T}_{3_{400}}=0$. (**d**) Comparison between the number of infected neighbors and each node’s degree for all variants. The infection spread numbers are $\rho _{1}=0.0001$, $\rho _{2}=0$, and $\rho _{3}=0$ respectively.

The black histogram in Fig. 16 is the same as the blue histogram. There is no hub in this network. The dynamic of the epidemic process is shown in Fig. 17a. The figure is the time evolution of the number of individuals of all states (S, I, R) with $95\%$ confidence interval. Here, we still divide this scenario into four cases.

For the first case of Scenario 4, we set $\mu _{_{1 \rightarrow 2}}\sim \text {Bernoulli}(0.005), \; \mu _{_{2 \rightarrow 3}}\sim \text {Bernoulli}(0.005)$ and $\beta _{1}=\beta _{2}=\beta _{3}=0.01$. The number of infected individuals from all variants indicated by the red line is shown in Fig. 17a. The emergence of new variants is shown in Fig. 17b. The figure is the average plot of 100 samples. The infection peak of Variant 1 is 8 nodes at the time $t=16$. There is no spread of Variant 2 and Variant 3. The spread of Variant 1 ends at time $t=39$. We can see the final value of the total number of infected individuals for each variant in Fig. 17c. From the total number of infected individuals of Variant 1 $AI^{T}_{1_{400}}=11$ and the length of epidemic time of Variant 1 is $t_{e_{1}}=38$ days, we can find the infection spread number of Variant 1 $\rho _{1}=0.0001$. We can compare the number of infected neighbors to the degree of each node in Fig. 17d.

**Figure 18:**
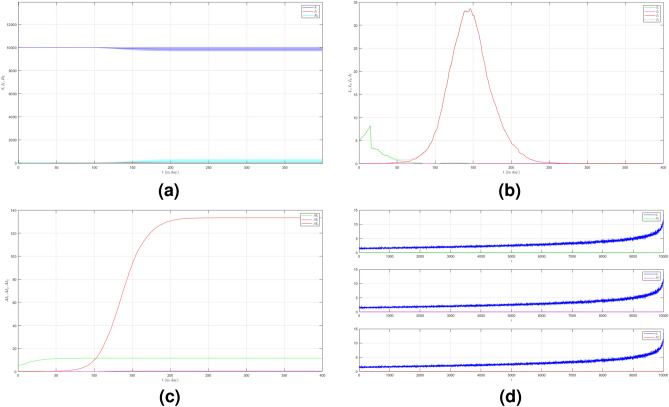
The dynamics of the epidemic process with three variants on a synthesized human interaction network for the second case of Scenario 4. (**a**) The time evolution of all states with $95\%$ confidence interval. The red lines indicate all infected nodes from all variants. (**b**) The average of 100 infection plots for each variant. The infection peaks of all variants are indicated by $I_{1_{16}}=9$, $I_{2_{121}}=1$, and $I_{3_{147}}=34$ respectively. (**c**) The total number of infections for all variants are $AI^{T}_{1_{400}}=12$, $AI^{T}_{2_{400}}=1$, and $AI^{T}_{3_{400}}=134$. (**d**) Comparison between the number of infected neighbors and each node’s degree for all variants. The infection spread numbers are $\rho _{1}=0.0001$, $\rho _{2}=0$, and $\rho _{3}=0.005$ respectively.

In the second case of Scenario 4, we still set $\mu _{_{1 \rightarrow 2}}\sim \text {Bernoulli}(0.005), \; \mu _{_{2 \rightarrow 3}}\sim \text {Bernoulli}(0.005)$ but $\beta _{1}<\beta _{2}<\beta _{3}$. We still choose $\beta _{1}=0.01, \; \beta _{2}=0.02$ and $\beta _{3}=0.03$ to show how different infection rates play roles in the epidemic process when the two containment measures are implemented. The dynamic of the epidemic process in this case is shown in Fig. 18a. There is an increase in the number of infections due to Variant 3 as shown in Fig. 18b. The infection peak of Variant 1 is 9 nodes at time $t=16$. There is no spread of Variant 2. The infection peak of Variant 3 is 34 at time $t=147$. Variant 1 ends at time $t=47$. Variant 3 starts to spread at time $t=76$ and ends at time $t=225$. The final value of the total number of infected individuals for each variant is shown in Fig. 18c. From the total number of infected individuals of Variant 1 $AI^{T}_{1_{400}}=12$ and the length of epidemic time $t_{e_{1}}=46$ days, we can find the infection spread number of Variant 1 $\rho _{1}=0.0001$. Since there is no spread of Variant 2, we write $\rho _{2}=0$. For Variant 3, we obtain $AI^{T}_{3_{400}}=134$ with $t_{e_{3}}=149$. We obtain $\rho _{3}=0.005$. In this case, reducing the connectivity and the frequency of contact is not strong enough to contain the spread of variants if the infection rate of the new variant is more infectious.

**Figure 19:**
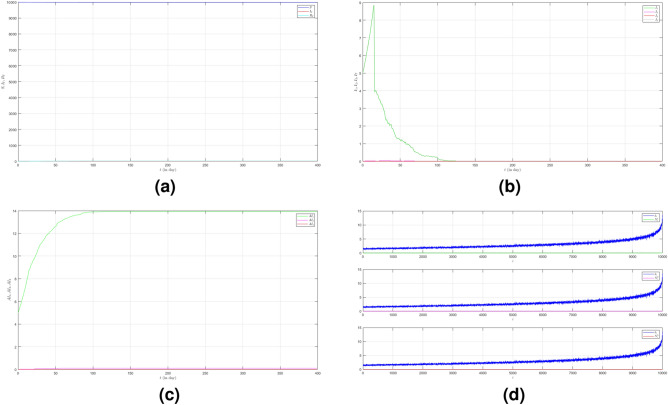
The dynamics of the epidemic process with three variants on a synthesized human interaction network for the third case of Scenario 4. (**a**) The time evolution of all states with $95\%$ confidence interval. The red lines indicate all infected nodes from all variants. (**b**) The average of 100 infection plots for each variant. The infection peaks of Variant 1s is $I_{1_{16}}=9$. (**c**) The total number of infections for all variants are $AI^{T}_{1_{400}}=14$, $AI^{T}_{2_{400}}=0$, and $AI^{T}_{3_{400}}=0$. (**d**) Comparison between the number of infected neighbors and each node’s degree for all variants. The infection spread numbers are $\rho _{1}=0.0002$, $\rho _{2}=0$, and $\rho _{3}=0$ respectively.

Now we set the third case of Scenario 3 where $\mu _{_{1 \rightarrow 2}}\sim \text {Bernoulli}(0.001), \; \mu _{_{2 \rightarrow 3}}\sim \text {Bernoulli}(0.001)$ and $\beta _{1}=\beta _{2}=\beta _{3}=0.01$. In this case, we want to see what happens if we reduce the probability of the emergence of new variants in the network with reduced connectivity and frequency of contact. The dynamic of the epidemic process in this case is shown in Fig. 19a. There is no infection by Variant 2 and Variant 3 as shown in Fig. 14b. The infection peak of Variant 1 is 9 nodes at the time $t=16$. There is no spread of Variant 2 and Variant 3. Thus $\rho _{2}=\rho _{3}=0$. Variant 1 ends at time $t=59$. The total number of infected individuals of Variant 1 $AI^{T}_{1_{400}}=14$ and the epidemic duration $t_{e_{1}}=58$. We have $\rho _{1}=0.0002$.

**Figure 20:**
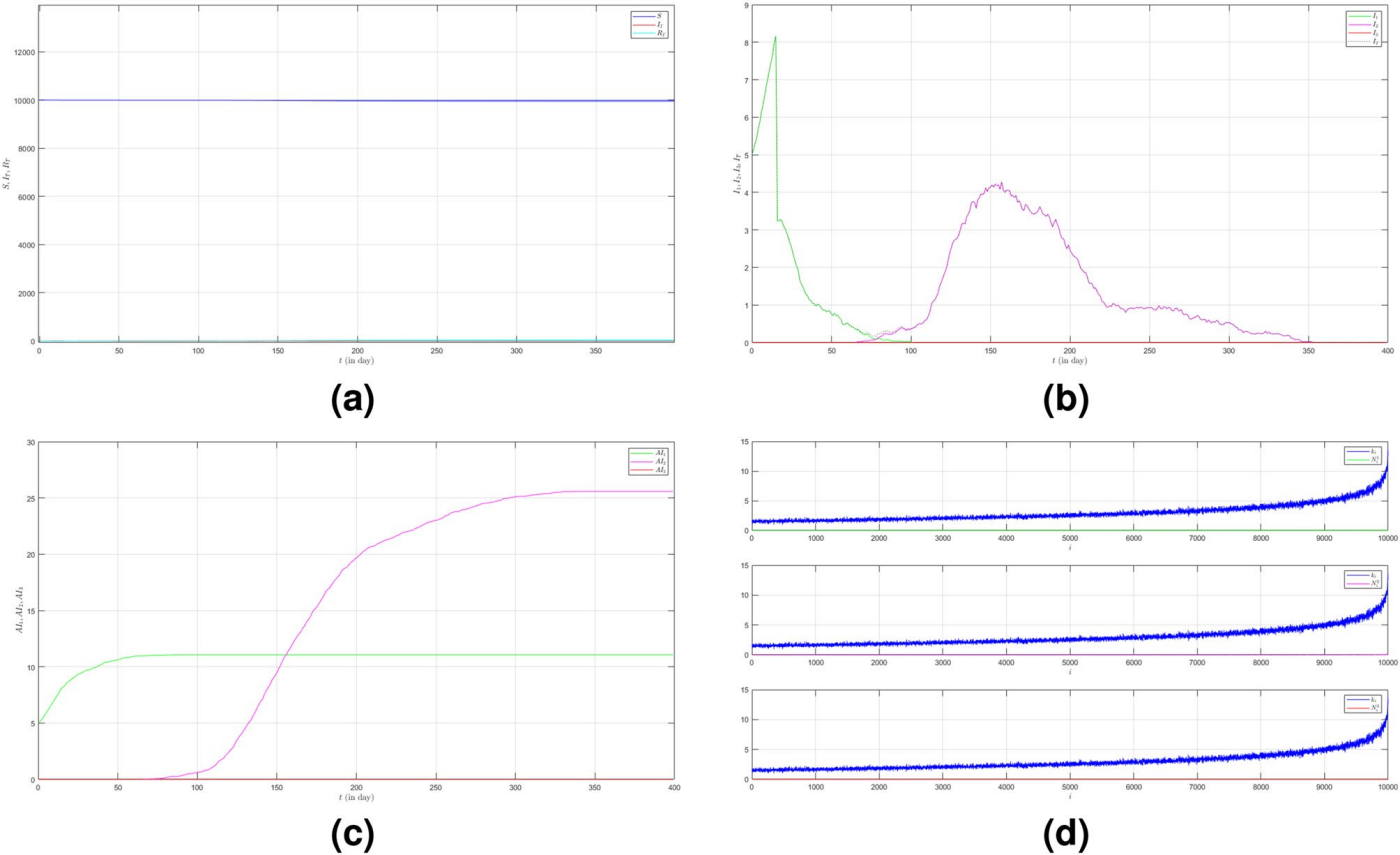
The dynamics of the epidemic process with three variants on a synthesized human interaction network for the fourth case of Scenario 4. (**a**) The time evolution of all states with $95\%$ confidence interval. The red lines indicate all infected nodes from all variants. (**b**) The average of 100 infection plots for each variant. The infection peaks of Variant 1 and Variant 2 are $I_{1_{16}}=9$ and $I_{2_{158}}=614$. (**c**) The total number of infections for all variants are $AI^{T}_{1_{400}}=12$, $AI^{T}_{2_{400}}=26$, and $AI^{T}_{3_{400}}=0$. (**d**) Comparison between the number of infected neighbors and each node’s degree for all variants. The infection spread numbers are $\rho _{1}=0.0001$, $\rho _{2}=0.0007$, and $\rho _{3}=0$ respectively.

To see how different infection rates from each variant play roles in a condition when the probability of the emergence of new variants is smaller and all contained measures are implemented, we set the fourth case of Scenario 3 where $\mu _{_{1 \rightarrow 2}}\sim \text {Bernoulli}(0.001), \; \mu _{_{2 \rightarrow 3}}\sim \text {Bernoulli}(0.001)$ and $\beta _{1}<\beta _{2}<\beta _{3}$. We still choose $\beta _{1}=0.01, \; \beta _{2}=0.02$ and $\beta _{3}=0.03$. The dynamic of the epidemic process in this case is shown in Fig. 20a. The time evolution of infected individuals for each variant is shown in Fig. 20b. The infection peak of Variant 1 is 9 nodes at time $t=16$. The infection peak of Variant 2 is 5 nodes at time $t=158$. There is no infection of Variant 3. Variant 1 ends at time $t=41$. Variant 2 starts to spread at time $t=114$ and ends at time $t=224$. The total number of infected individuals for each variant is shown in Fig. 20c. The total number of infected individuals of Variant 1 $AI^{T}_{1_{400}}=12$ and the the length of epidemic time of Variant 1 $t_{e_{1}}=40$ days give the infection spread number of Variant 1 $\rho _{1}=0.0001$. For Variant 2 we obtain $AI^{T}_{2_{400}}=26$ with $t_{e_{2}}=110$. We have $\rho _{2}=0.0007$.

## Discussion

We developed a computational model of disease spreading with three variants that can capture the microscopic process in a synthesized human interaction network. In generating the network we need some social features in human interaction such as connectivity and contact frequency or interaction intensity. Both features determine the network structures such that every individual has an unequal chance of coming into contact with any other individual in the population. Of course, it is more realistic to describe a human population although not a real representation of the social network of the human population. Therefore, discussing the spread of contact-based diseases in a human population is more relevant in smaller-scale environments. However, we need to develop some critical aspects. Because it is a microscopic process and we brought the model to an individual scale, as a consequence, unlikely the classical SIR model, our model does not use merely infection rate $\beta$. we also do not use a recovery rate as usual we use it in the common SIR model. Incorporating more epidemiological parameters such as the average duration of the infectious period, the probability of the emergence of new variants, the length of the infection period, the length of the incubation period, and many more, has also consequences. In our model, we perform some tricks to the parameters such that they can be incorporated into the model and more applicable for both mathematics and computation purposes. We can also generalize our model to any number of variants and incorporate more epidemiological parameters such that the model is more complicated and realistic. Hopefully, there will be a study to analyze our model rigorously.

Our model has rich computational results by varying several parameters such as the number of individuals in the network, the average degree, the range of contact frequency, the values of epidemiological parameters, the initial conditions, the probability of the emergence of the new variants, and the infection rate of each variant. In the simulation, we chose to vary the average degree and contact frequency to elucidate the role of social network structures in disease spreading by keeping several epidemiological parameters and initial conditions constant. By varying the average degree and contact frequency we also address the containment measures implemented into the network to contain the spreads. The simulation results gave us a particular insight into how the social network structures determine epidemic behaviors such as the peak values of infection curves, the time to reach them, the increasing and decreasing rate of the infection curves, the infection spread numbers, and the final size of the epidemic. Here, we try to describe the disease spreading between the population that implements the containment measures and the one that does not. Next, from the model, we can develop a model that incorporates more interventions to derive efficient and realistic strategies for curbing the spread of disease throughout the network.

## Method

The first step to generate a synthesized human interaction network with size *N* and an average degree ${\bar{k}}$ is using the simplest examples of a random network in which we fix only the number of nodes *N* and the number of edges $L={\bar{k}}N/2$. We take *N* nodes and place *L* edges among them at random^19^. The next step is to attach each edge with a natural number which is randomly chosen from set $W=\left\{ 1,2,\dots ,\omega \right\} \subseteq {\textbf{N}}$, where $\omega$ is the maximum of contact frequency. Here, we obtain the weighted adjacency matrix ${\textbf{A}}=\left( \omega _{ij}\right)$ with $\omega _{ij} \in W \cup \left\{ 0\right\}$ to represent the network. We assume that the network is undirected. If $\omega _{ij}=0$, then there is no interaction or link between node *i* and node *j*. From ${\textbf{A}}$, we can obtain $N_{i}$ as a set containing all neighbors of node *i*.

To simplify the discrete-time process we assume that one day is a unit time step and we write $t=0,1,2,\dots ,t_{f}$ as a time discretization. We denote $t_{f}$ as the duration of the epidemic process. At time *t*, a node *i* can be in a state $X^{i}_{_t}$ belonging to a finite set of states $\Sigma =\left\{ S^{i}_{_{t}},I^{i}_{1_{t}}, R^{i}_{1_{t}}, I^{i}_{2_{t}}, R^{i}_{2_{t}},I^{i}_{3_{t}}, R^{i}_{3_{t}}\right\}$. The state $X^{i}_{_{t}} \in \left\{ 0,1 \right\}$ and

1 $$\begin{aligned} S^{i}_{_{t}}+I^{i}_{1_{t}}+ R^{i}_{1_{t}}+ I^{i}_{2_{t}}+R^{i}_{2_{t}}+I^{i}_{3_{t}}+ R^{i}_{3_{t}}=1 \; \text { for all nodes } i \text { and for all time } t \end{aligned}$$

Since we put three variants into the network, we write $v=1,2,3$ to denote the variants, and the original virus is Variant 1. All states are explained in the following table (Table 1).

**Table 1:** The Description of states.

State	Description
$S^{i}_{_{t}}$	State at time *t* when a node *i* is susceptible ($S^{i}_{_{t}}=1$) or not ($S^{i}_{_{t}}=0$)
$I^{i}_{v_{t}}$	State at time *t* when a node *i* is infected by Variant *j* ($I^{i}_{v_{t}}=1$) or not ($I^{i}_{v_{t}}=0$)
$R^{i}_{v_{t}}$	State at time *t* when node *i* is recovered from Variant *j* ($R^{i}_{v_{t}}=1$) or not ($R^{i}_{v_{t}}=0$)

To introduce the infections due to the Variant 1 at the initial time, we choose randomly $N_{0}$ individuals. Variant 2 will emerge from Variant 1 by moving the infected individual in the state $I^{i}_{1_{t}}$ to the state $I^{i}_{2_{t}}$ which is denoted by a Bernoulli random variable $\mu _{_{1 \rightarrow 2}}$ with a small probability. Variant 3 will emerge from Variant 2 with a similar procedure. The infected individual in the state $I^{i}_{2_{t}}$ will move to the state $I^{i}_{3_{t}}$ which is denoted by a Bernoulli random variable $\mu _{_{2 \rightarrow 3}}$ with a small probability.

**Figure 21:**
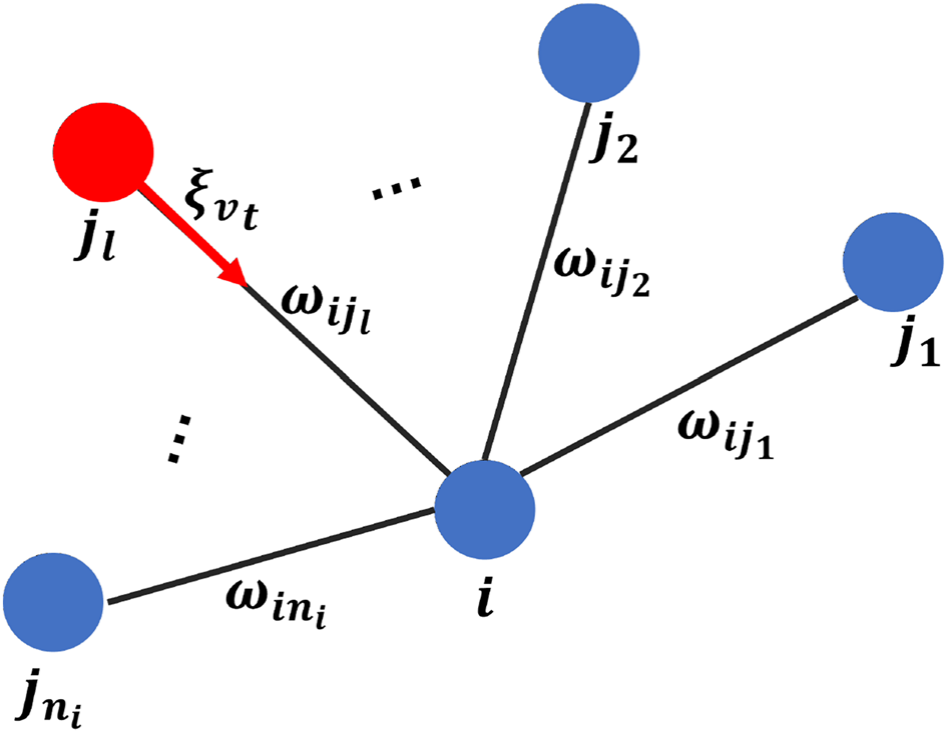
The viral transmission to node *i* from its neighbours. The red node represents an infected one. The blue nodes can be susceptible or recovered from previous variants.

For each variant $v=1,2,3$, we let $\xi _{v_{t}}$ as a Bernoulli random variable with a probability $\beta _{v}$ to denote viral transmission between two nodes. We define that $\beta _{v}$ is the average rate of infection in one contact for Variant *v*. The process of viral transmission from one of the infected neighbors of node *i* to node *i* can be illustrated in Fig. 21. The viral transmission to node *i* at time *t* for each variant can be expressed by the following equation.

2 $$\begin{aligned} \phi ^{i}_{v_{t}} = 1-\prod _{j\in N_{i}}\prod _{k=1}^{\omega _{ij}}\left( 1-\xi _{v_{t}}I^{j}_{v_{t-1}}\right) \end{aligned}$$

Note that the Eq. (2) describes how viral transmission occurs at the individual level or microscopic scale. Not only does the equation consider all neighbors for each individual but also considers the frequency of contact with all neighbors. It also tells us that the bigger the number of neighbors and weight, the more likely a node *i* to get an infection.

To make clear when the neighbors of node *i* contain the infected individuals with more than one variant, the viral transmission to node *i* occurs when the first infected individual succeeds in transmitting the infection. With this rule, we avoid an individual getting more infections at one time. Thus, we write

3 $$\begin{aligned} \phi ^{i}_{1_{t}}+\phi ^{i}_{2_{t}}+\phi ^{i}_{3_{t}}=1 \; \text { for\;all\;node }\;i\;\text {and\;for\;all\;time }\;t \end{aligned}$$

We assume that each individual will gain permanent immunity after she or he recovers from one variant but lose immunity if she or he gets infected by other variants.

**Figure 22:**
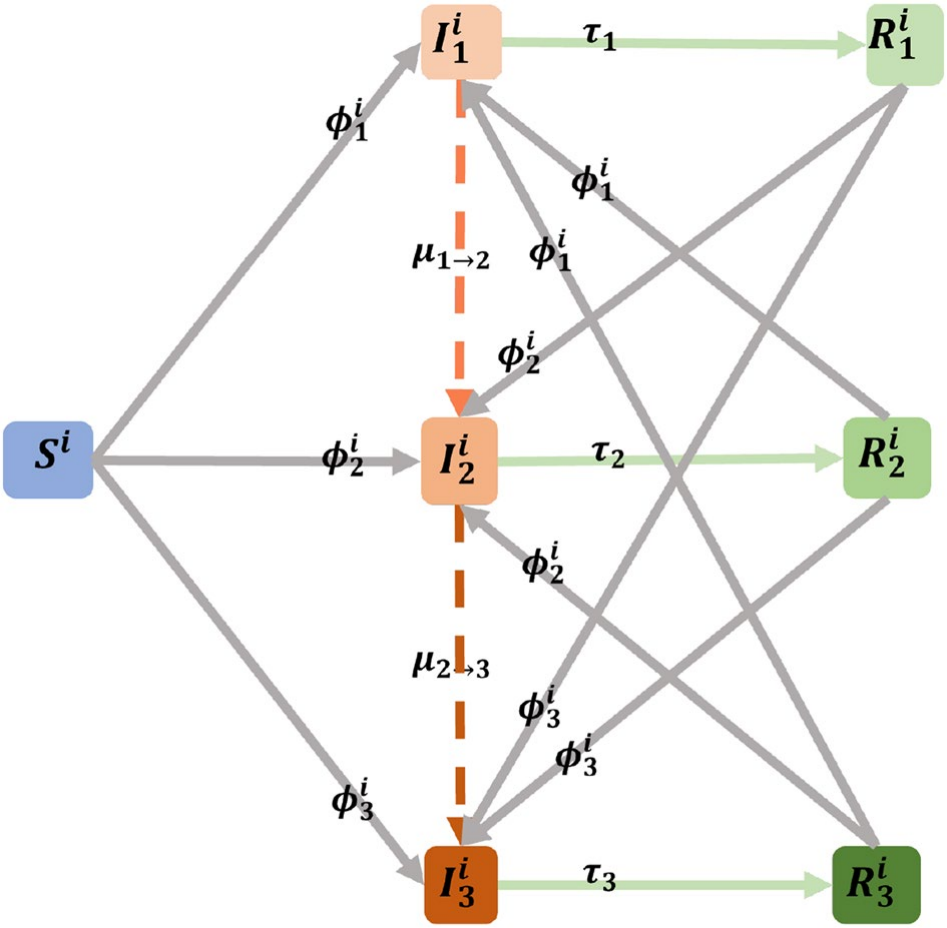
The process of all possibilities of state transition for each node in the network at a single time *t*.

Unlike the common SIR model, we do not use the recovery rate. Instead, we use the parameters $t^{i}_{v}$, $\tau _{v}$ and the unit-step functions $u\left( t,t^{i}_{v}\right)$ and $u\left( t,t^{i}_{v}+\tau _{v}\right)$ for each variant *v*. The parameter $t^{i}_{v}$ is the time when a node *i* gets infected by Variant *v* for the first time. Meanwhile, the parameter $\tau _{v}$ is the average duration of the infectious period for each variant. The functions $u\left( t,t^{i}_{v}\right)$ and $u\left( t,t^{i}_{v}+\tau _{v}\right)$ will move the individual from the susceptible state to the infected state and from the infected state to the recovery state respectively. The process of state transition for each node in the network is shown in Fig. 22 and the process is governed by the following system of equations.

4 $$\begin{aligned} S^{i}_{t+1}&= S^{i}_{t}\left( 1-\left( \phi ^{i}_{1_{t+1}}+\phi ^{i}_{2_{t+1}}+\phi ^{i}_{3_{t+1}}\right) \right) \end{aligned}$$

5 $$\begin{aligned} I^{i}_{1_{t+1}}&= I^{i}_{1_{t}}\left( 1-u\left( t, t^{i}_{1}+\tau _{1}\right) -\mu _{_{1 \rightarrow 2}}\right) + \phi ^{i}_{1_{t+1}} \left( S^{i}_{t} + R^{i}_{2_{t}} +R^{i}_{3_{t}} \right) \end{aligned}$$

6 $$\begin{aligned} I^{i}_{2_{t+1}}&= I^{i}_{2_{t}}\left( 1-u\left( t, t^{i}_{2}+\tau _{2}\right) -\mu _{_{2 \rightarrow 3}}\right) +\mu _{_{1 \rightarrow 2}}I^{i}_{1_{t}}+ \phi ^{i}_{2_{t+1}} \left( S^{i}_{t} + R^{i}_{1_{t}} +R^{i}_{3_{t}} \right) \end{aligned}$$

7 $$\begin{aligned} I^{i}_{3_{t+1}}&= I^{i}_{3_{t}}\left( 1-u\left( t, t^{i}_{3}+\tau _{3}\right) \right) +\mu _{_{2 \rightarrow 3}}I^{i}_{2_{t}} + \phi ^{i}_{3_{t+1}} \left( S^{i}_{t} + R^{i}_{1_{t}} +R^{i}_{2_{t}} \right) \end{aligned}$$

8 $$\begin{aligned} R^{i}_{1_{t+1}}&= R^{i}_{1_{t}}\left( 1-\left( \phi ^{i}_{2_{t+1}}+\phi ^{i}_{3_{t+1}}\right) \right) +u\left( t, t^{i}_{1}+\tau _{1}\right) I^{i}_{1_{t}} \end{aligned}$$

9 $$\begin{aligned} R^{i}_{2_{t+1}}&= R^{i}_{2_{t}}\left( 1-\left( \phi ^{i}_{1_{t+1}}+\phi ^{i}_{3_{t+1}}\right) \right) +u\left( t, t^{i}_{2}+\tau _{2}\right) I^{i}_{2_{t}} \end{aligned}$$

10 $$\begin{aligned} R^{i}_{3_{t+1}}&= R^{i}_{3_{t}}\left( 1-\left( \phi ^{i}_{1_{t+1}}+\phi ^{i}_{2_{t+1}}\right) \right) +u\left( t, t^{i}_{3}+\tau _{3}\right) I^{i}_{3_{t}} \end{aligned}$$

To indicate whether a node *i* has been infected, we use the following equations.

11 $$\begin{aligned} AI^{i}_{1_{t+1}}&= AI^{i}_{1_{t}}+\phi ^{i}_{1_{t+1}} \left( S^{i}_{t} + R^{i}_{2_{t}} +R^{i}_{3_{t}} \right) \end{aligned}$$

12 $$\begin{aligned} AI^{i}_{2_{t+1}}&= AI^{i}_{2_{t}}+\phi ^{i}_{2_{t+1}} \left( S^{i}_{t} + R^{i}_{1_{t}} +R^{i}_{3_{t}} \right) \end{aligned}$$

13 $$\begin{aligned} AI^{i}_{3_{t+1}}&= AI^{i}_{3_{t}}+\phi ^{i}_{3_{t+1}} \left( S^{i}_{t} + R^{i}_{1_{t}} +R^{i}_{2_{t}} \right) \end{aligned}$$

Based on Eqs. (4)–(10), we can calculate the number of nodes for each state at time *t* with the following equations.

14 $$\begin{aligned} S_{t}=\sum _{i=1}^{N} S^{i}_{t},\; I_{v_{t}}=\sum _{i=1}^{N} I^{i}_{v_{t}},\; R_{v_{t}}=\sum _{i=1}^{N} R^{i}_{v_{t}}, \; v=1,2,3 \end{aligned}$$

The final number of infections for each variant at the final time can be calculated by the following equations.

15 $$\begin{aligned} AI^{T}_{1_{}}=\sum _{i=1}^{N} AI^{i}_{1_{t}},\; AI^{T}_{2_{t}}=\sum _{i=1}^{N} AI^{i}_{2_{t}},\; AI^{T}_{3_{t}}=\sum _{i=1}^{N} AI^{i}_{3_{t}} \end{aligned}$$

In epidemiology, we used to consider basic reproduction number ${\mathscr {R}}_{0}$ to describe the ability of the disease to spread in a population. It is an average number of additional infected individuals produced by such individual passes the disease onto before it recovers^19^. Referring to the classic SIR model, when ${\mathscr {R}}_{0}>1$, infected individuals will grow geometrically, causing an epidemic^26^. When ${\mathscr {R}}_{0}<1$, the disease will die out. It can be calculated straightforwardly by finding the ratio of infection and recovery rate. However, it is not easy to find the number from our model. We do not have a recovery rate for each variant. In addition, our model uses three variants and relies on the microscopic process in discrete time. We consider the epidemic process on an individual scale. The size of the population is limited to a certain number which is much smaller than the size of the population in the common SIR model. Trying to find the basic reproduction number of the model in this research is no longer relevant. Thus, we do not deal with the basic reproduction number for our model because the model we developed is fully different from the common SIR model. Instead of using it, we define a number that represents how big and long an epidemic spreads. We call the number the infection spread number and denote it with $\rho$. We define it explicitly for each variant as the following.

16 $$\begin{aligned} \rho _{v}=\frac{AI^{T}_{v_{t_{f}}}\;t_{e_{v}}}{N\;t_{f}}, \; v=1,2,3 \end{aligned}$$

where $t_{e_{v}}$ is the length of epidemic time which is defined as when the infection starts to spread and when it ends. The infection spread number has no dimension. We can interpret the number to infer how severe the epidemic is. The larger its value takes, the bigger and the longer, the size and duration of the epidemic will be.

## Data Availability

The datasets generated and/or analyzed during the current study are available in the https://github.com/seprianus9981/seprianus_paper/blob/main/simvarian3.tex.
